# Endovascular Stenting in Superior Vena Cava Syndrome: A Systematic Review and Meta-analysis

**DOI:** 10.1007/s00270-022-03178-z

**Published:** 2022-07-12

**Authors:** Eri Yin-Soe Aung, Maha Khan, Norman Williams, Usman Raja, Mohamad Hamady

**Affiliations:** 1 Imperial College School of Medicine, Imperial College London, Sir Alexander Fleming Building, Imperial College Road, London, SW72DD UK; 2 Surgical and Interventional Trials Unit (SITU), University College London, London, W1W 7JN UK; 3 Imperial College Healthcare NHS Trust, St Mary’s Hospital, London, UK; 4 Guy’s and St Thomas NHS Trust, London, UK

**Keywords:** Endovascular stenting, Superior vena cava syndrome, Systematic review, Meta-analysis

## Abstract

**Purpose:**

Endovascular stenting has been used to manage superior vena cava syndrome for several decades and has become standard firstline practice. This study aims to investigate the outcomes of endovascular stenting in the management of superior vena cava syndrome (SVCS).

**Methods:**

MEDLINE, EMBASE and PUBMED online databases were searched, with studies involving more than ten adult patients included. Studies identified spanned 27 years, from 1993 to 2020. Meta-analyses were performed based on Clopper–Pearson estimation.

**Results:**

Fifty-four studies were identified, for a total of 2249 patients, of which 2015 had malignant SVCS and 222 benign SVCS. Pooled technical success and clinical success rates were 96.8% (95% CI 96.0–97.5%) and 92.8% (95% CI 91.7–93.8%). Technical success and clinical success rates for studies investigating benign SVCS alone were identical at 88.8% (95% CI 83.0–93.1%). Pooled patency remained above 90% for the first year. Average complication and re-intervention rates were 5.78% (SD = 9.3182) and 9.11% (SD = 11.190).

**Conclusions:**

This review confirms the effectiveness of endovascular stenting in managing SVCS. Further directions of research may include specific outcomes of endovascular stenting in benign SVCS, and the impact of procedural characteristics, such as the use of anticoagulation and type of stent used, on outcomes.

**Level of Evidence:**

Level III, systematic review of retrospective cohort studies.

## Introduction

Superior vena cava syndrome (SVCS) arises when the superior vena cava (SVC) becomes partially or completely obstructed. Depending on the speed of onset, allowing the development of venous collaterals over time, the symptomology of SVCS ranges from asymptomatic to minor symptoms (e.g. headache, cough or neck vein distension), to acute respiratory compromise and rarely, mortality from laryngeal or cerebral oedema [1–3].

The aetiology of SVCS is predominantly due to malignant obstruction, with either primary malignancies or lymph node metastases extrinsically compressing or directly invading the SVC [1, 4]. SVCS is however increasingly caused by benign pathologies. Indwelling intravascular catheters or cardiac device leads have replaced rarer pathologies such as fibrosing mediastinitis to become the commonest benign cause of SVCS [4–10].

Traditional treatment modalities for malignant SVCS include radiotherapy, chemotherapy and surgical bypass [1, 4, 11]. The use of stenting as first-line therapy has gathered popularity to become standard practice in the past two decades [4, 10]. Endovascular intervention has been associated with more rapid, complete symptom relief and lower complication rates [1, 4, 12]. It also provides greater flexibility, as attempting subsequent alternative therapies is not precluded [10, 12]. Evidence is however limited primarily to single-centre studies and the impact of procedural characteristics such as stent type or use of anticoagulation has not been thoroughly explored.

This study aims to consolidate and summarise the published literature about the outcomes of endovascular stenting in SVCS via means of a systematic review and meta-analysis. We aim to synthesize the current evidence regarding outcomes including technical success of the procedure, clinical symptom resolution and reported recurrence and complications, as well as provide a comprehensive overview of the impact of procedural characteristics on these outcomes.

## Methods

This systematic review and meta-analysis was designed and performed according to the Preferred Reporting Items of Systematic Reviews and Meta-Analyses (PRISMA) standards [13]. Study methodology was specified prior to data extraction and registered with PROSPERO (CRD 42021191795).

### Literature Search

Two authors (EA, MK) performed the search of MEDLINE, EMBASE and PUBMED online databases to identify articles related to the outcomes of endovascular stenting in the treatment of SVCS. The following search terms were used, alone and in combinations; “superior vena cava syndrome”, “superior vena cava obstruction”, “superior vena cava” and “stent”. All retrieved studies were first screened on title and abstract, then screened studies read in full by both authors to determine eligibility for inclusion. The final search was on 14^th^ November 2020.

### Study Selection

Study selection was performed independently by two authors (EA, MK). The selection criteria were as follows: (1) Full text of the study had to be available in English. (2) Studies had to include 10 or more adult human patients. (3) Where studies concerned interventions in the SVC as well as other vessels, only studies with identified data for technical and clinical outcomes of SVC interventions, with or without involvement of brachiocephalic veins, were included.

### Data Collection and Quality Assessment

The following data were extracted from each included study: (1) details of the study—first author, year, study type (prospective/retrospective), journal of publication, conflict of interests; (2) population demographic data—size of study population, mean age, gender, benign or malignant pathology, pre-intervention chemotherapy or radiotherapy; (3) procedural data—type and make of stent, use of anticoagulation or thrombolysis, technical and clinical success, complications, and (4) follow-up data—primary and secondary patencies, recurrence of symptoms, re-interventions and survival. Data were extracted independently by two authors (EA, MK). Where the reviewers had any disagreement, this was resolved by discussion and where necessary, consensus with the senior author (MH). The methodological quality of the included studies was assessed for risk of bias using the Newcastle–Ottawa scale [14].

### Statistical Analysis

Meta-analyses were performed to report technical and clinical success of stenting to relieve SVCS, as well as recurrence of symptoms at 1, 3, 6 and 12 months. Using a random effects model, individual and pooled proportions and 95% confidence intervals were calculated by the Clopper–Pearson estimation method based on the exact binomial distribution. Statistical heterogeneity was assessed using the *I*^2^ (inconsistency) statistic. SAS software version 9.4 was used for analysis and production of the graphs.

## Results

### Study Selection

**Fig. 1:**
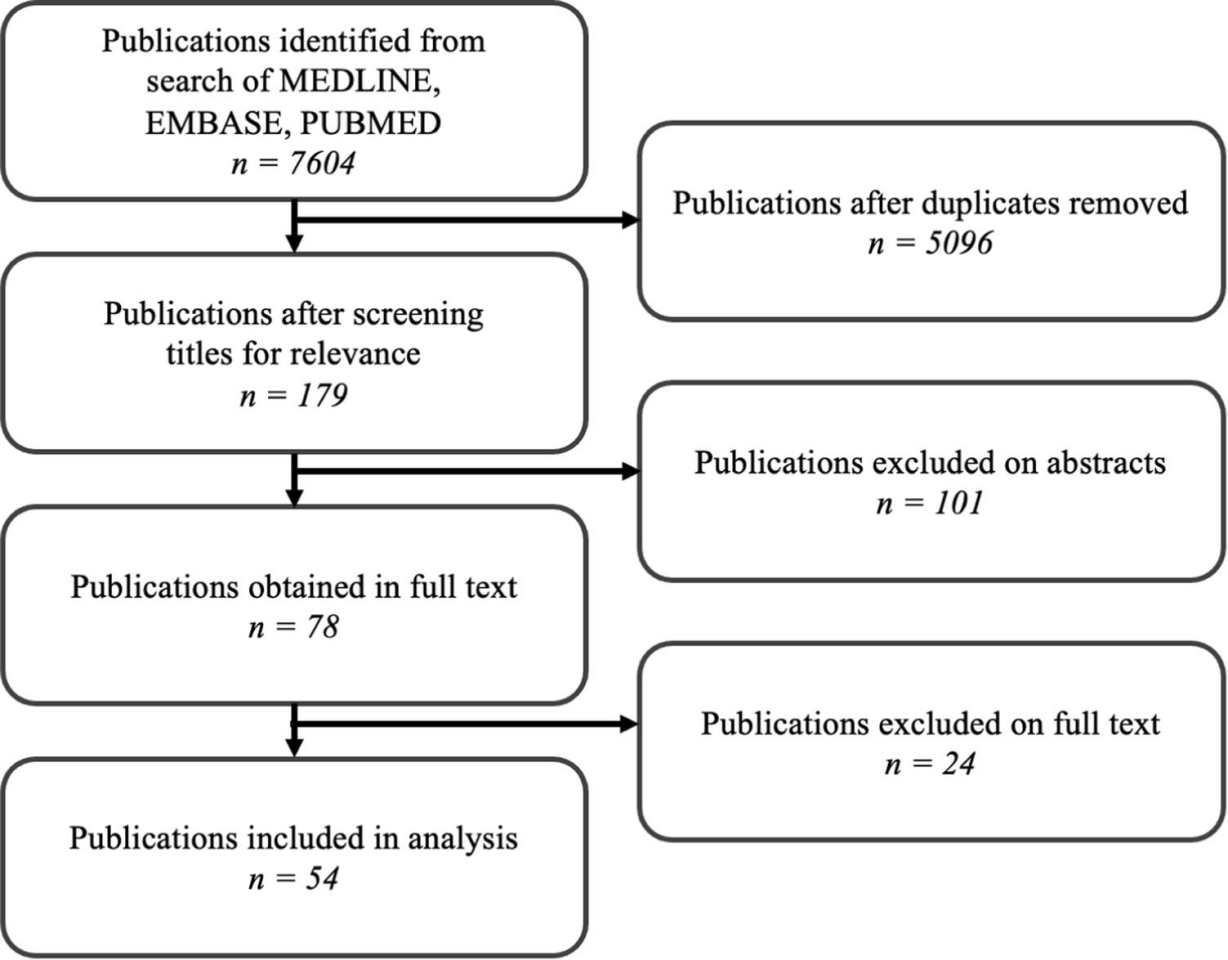
PRISMA flowchart showing selection of studies for analysis. Selection criteria were as follows: (1) Full text of the study had to be available in English. (2) Studies had to include 10 or more adult human patients. (3) Where studies concerned interventions in the SVC as well as other vessels, only studies with identified data for technical and clinical outcomes of SVC interventions, with or without involvement of brachiocephalic veins, were included

The initial search resulted in 7604 studies (Fig. 1). After removal of duplicates and screening on title and abstract, 78 studies were obtained and read in full text, of which 54 met all inclusion criteria, for a total of 2249 patients. Data extraction and study quality assessment were subsequently performed. The most frequent reasons for exclusion were insufficient sample sizes or undifferentiated reporting of outcomes in the SVC.

### Patient Demographics and Study Characteristics

The total number of patients reported was 2249. The cumulative mean age of all patients was 58.7 years and the sex ratio (males/females) was 2.6 (1605/612). One study presented no data on patient demographics [15], while two did not present mean age [16, 17].

Characteristics of included studies are summarised in Table 1. Of the 54 studies included, 34/54 were retrospective, 17/54 were prospective, and 3/54 had both prospective and retrospective arms. No randomised controlled trials or multi-centre studies were identified. The risk of conflict of interests in all studies was low. Risk of bias, assessed via the Newcastle–Ottawa scale, is shown in Table 2. All papers scored between 7 and 9, indicating high quality.

**Table 1:** Characteristic of the 54 included studies, including number of patients, mean age (years), pathology of SVCS studied, whether patient groups had received previous therapies for malignant SVCS, vessels involved, technical success rate, clinical success rate, pre-operative assessment in diagnosing SVCS, stent details and brands, use of procedural anticoagulation or antiplatelet therapy and follow-up protocol

	No. of patients (*n*)	Mean age (years)	Patient characteristics	Vessels involved	Tech. success (%)	Clin. success (%)	Pre-operative assessment	Stent details	Procedural details	Follow-up Protocol
Dyet et al. [23]	17	63.4	Malignant: 17 (100%) CRT: 0, C: 0, R: 14	SVC: 17 (100%) + BCV: 6 (35%) + IVC: 1 (6%)	100	100	CT thorax Histology Venography	Uncovered: 17 (100%) Wallstent	Anticoag: Heparin 5000 IU Warfarin 3 m	Imaging: Venogram at 1 m, 3 m Clinical: Patient reported Mean F/U: NR
Gaines et al. [15]	20	NR	Malignant: 20 (100%) CRT: 0, C: 5, R: 11	NR	90	90	Venography	Uncovered: 20 (100%) Gianturco-Z	Anticoag: Heparin 5d	Imaging: NR Clinical: Patient reported Mean F/U: NR
Crowe et al. [35]	13	55.5	Malignant: 12 (92%) CRT: 0, C: 1, R: 10 Benign: 1 (8%)	SVC: 13 (100%) + BCV: 11 (85%)	84.6	84.6	Venography	Uncovered: 11 (100%) Gianturco-Z, Wallstent, Palmaz	Anticoag: Heparin 5d	Imaging: NR Clinical: Patient reported Mean F/U: NR
Hennequin et al. [57]	14	60	Malignant: 13 (93%) CRT: 8, C: 5, R: 0 Benign: 1 (7%)	SVC: 14 (100%) + BCV: 9 (64%)	100	92.9	CT thorax Venography	Uncovered: 14 (100%) Wallstent	Anticoag: Heparin 5000 IU Heparin 24 h, LMWH 1 m	Imaging: CT at 3 m, 6 m Clinical: Patient reported Mean F/U: 4.1 m
Shah et al. [58]	13	60	Malignant: 13 (100%) CRT: 0, C: 0, R: 2	NR	92.3	84.6	Histology Venography	Uncovered: 12 (100%) Gianturco-Z, Wallstent	Anticoag: Heparin 5000 IU Heparin 2d	Imaging: NR Clinical: Patient reported Mean F/U: 3.7 m
Stock et al. [51]	14	62	Malignant: 14 (100%) CRT: 1, C: 7, R: 3	SVC: 14 (100%) + BCV: 9 (64%)	85.7	85.7	Venography	Uncovered: 12 (100%) Wallstent	Anticoag: Heparin 5000 IU	Imaging: NR Clinical: Patient reported Median F/U: 171d
Oudkerk et al. [18]	30	60.4	Malignant: 30 (100%) CRT: 0, C: 12, R: 22	NR	100	96.7	Venography	Uncovered: 30 (100%) Wallstent	Anticoag: Heparin	Imaging: Venogram at 2w Clinical: Patient reported Mean F/U: 2.5 m
Gross et al. [32]	13	60.2	Malignant: 13 (100%) CRT: 6, C: 0, R: 5	SCV: 13 (100%) + BCV: 4 (31%)	100	100	Venography	Uncovered: 13 (100%) Wallstent	Anticoag: Heparin Dual antiplatelets 4w	Imaging: NR Clinical: Patient reported Mean F/U: NR
Nicholson et al. [12]	81	62.2	Malignant: 81 (100%) CRT: 0, C: 8, R: 11	NR	93.8	93.8	Venography	Uncovered: 76 (100%) Wallstent	Anticoag: NR	Imaging: NR Clinical: Patient reported Mean F/U: NR
Tanigawa et al. [36]	23	61.2	Malignant: 23 (100%) CRT: 1, C: 0, R: 10	SVC: 23 (100%) + BCV: 6 (26%)	100	78.3	CT angiogram Venography	Uncovered: 23 (100%) Gianturco-Z	Anticoag: Heparin 3d	Imaging: NR Clinical: Patient reported Mean F/U: NR
Qanadli et al. [33]	12	54	Benign: 12 (100%)	SVC: 12 (100%) + BCV: 5 (42%)	100	100	CT thorax Venography	Uncovered: 12 (100%) Wallstent	Anticoag: Heparin 5000 IU Dual antiplatelets 4w	Imaging: CT at 3 m Clinical: Patient reported Mean F/U: 11 m
Thony et al. [37]	26	54	Malignant: 26 (100%) CRT: 5, C: 6, R: 0	SVC: 26 (100%) + BCV: 8 (30%)	96.2	80.8	CT thorax Venography	Uncovered: 25 (100%) Wallstent, Strecker	Anticoag: Heparin 3000 IU Aspirin 3 m	Imaging: CT at 6 m Clinical: Patient reported Mean F/U: NR
Marcy et al. [59]	39	59	Malignant: 37 (95%) CRT: NR, C: NR, R: NR Benign: 2 (5%)	NR	97.4	92.3	Venography	Uncovered: 39 (100%) Gianturco-Z, Strecker, Memotherm	Anticoag: Heparin 5000 IU Aspirin	Imaging: NR Clinical: Patient reported Mean F/U: 24w
Miller et al. [60]	23	64	Malignant: 23 (100%) CRT: 1, C: 0, R: 7	NR	100	82.6	CT thorax Venography	Uncovered: 23 (100%) Wallstent	Anticoag: NR	Imaging: NR Clinical: Patient reported Mean F/U: NR
Sasano et al. [29]	11	60	Malignant: 11 (100%) CRT: NR, C: NR, R: NR	SVC: 11 (100%) + BCV: 7 (64%)	100	90.9	CT thorax Venography	Uncovered: 11 (100%) Wallstent	Anticoag: Heparin 5000 IU Warfarin 3 m	Imaging: NR Clinical: Patient reported Mean F/U: NR
Lanciego et al. [19]	52	63	Malignant: 52 (100%) Stenting as first line intervention	SVC: 52 (100%) + BCV: 33 (63%)	100	100	Venography	Uncovered: 52 (100%) Wallstent	Anticoag: Heparin 1w Dual antiplatelets 6 m	Imaging: NR Clinical: Patient reported Mean F/U: NR
Smayra et al. [38]	30	61	Malignant: 16 (54%) CRT: 0, C: 0, R: 6 Benign: 14 (46%)	NR	100	100	Venography	Uncovered: 30 (100%) Memotherm, Wallstent, Symphony	Anticoag: Heparin 5000 IU	Imaging: NR Clinical: Patient reported Mean F/U: 10 m
Wilson et al. [70]	18	65	Malignant: 18 (100%) CRT: 0, C: 0, R: 6	SVC: 18 (100%) + BCV: 6 (33%)	100	100	Histology Venography	Uncovered: 18 (100%) Gianturco-Z, Strecker, Wallstent	Anticoag: NIL	Imaging: NR Clinical: Patient reported Mean F/U: NR
de Gregorio Ariza et al. [53]	82	57.8	Malignant: 68 (83%) CRT: NR, C: NR, R: NR Benign: 14 (17%)	NR	95.1	95.1	CT angiogram Venography	Uncovered: 82 (100%) Wallstent, Palmaz	Anticoag: Heparin 5000 IU	Imaging/Clinical: CXR/USS + assessment at 1, 3, 6, 12 m Mean F/U: 7 m (M), 31 m (B)
Chatziioannou et al. [61]	18	56.6	Malignant: 18 (100%) CRT: NR, C: NR, R: NR	SVC: 18 (100%) + BCV: 8 (44%)	100	100	CT thorax Histology Venography	Uncovered: 18 (100%) Memotherm	Anticoag: Heparin 5000 IU	Imaging: Venogram at 25d Clinical: Daily for 25d Mean F/U: NR
Courtehoux et al. [62]	20	58	Malignant: 20 (100%) CRT: 10, C: 9, R: 0	SVC: 20 (100%) + BCV: 5 (25%)	100	90	CT thorax	Uncovered: 20 (100%) Wallstent	Anticoag: Heparin 5000 IU Warfarin + aspirin	Imaging: NR Clinical: Patient reported Mean F/U: NR
Dinkel et al. [49]	84	64	Malignant: 84 (100%) CRT: 0, C: 54, R: 28	SVC: 84 (100%) + BCV: 71%	98.8	89.3	CT thorax Venography	Uncovered: 83 (100%) Wallstent	Anticoag: Heparin 5000 IU Long term anticoagulation	Imaging: NR Clinical: Patient reported Mean F/U: NR
Monaco (2003)	44	55.6	Malignant: 40 (91%) CRT: 33, C: 0, R: 0 Benign: 4 (9%)	SVC: 44 (100%) + BCV: 17 (39%)	100	100	CT thorax Venography	Uncovered: 44 (100%) Wallstent	Anticoag: Heparin 5000 IU Dual antiplatelets	Imaging: NR Clinical: Patient reported Mean F/U: NR
Kim et al. [63]	10	54	Malignant: 10 (100%) CRT: 5, C: 2, R: 1	NR	100	90	Venography	Uncovered: 10 (100%) Wallstent	Anticoag: Warfarin + aspirin	Imaging: NR Clinical: Patient reported Mean F/U: NR
Urreticoechea [30]	52	57	Malignant: 52 (100%) CRT: 4, C: 14, R: 2	NR	100	100	Histology Venography	Uncovered: 52 (100%) Wallstent, Memotherm	Anticoag: Heparin 5000 IU LMWH or warfarin 3 m	Imaging: NR Clinical: Patient reported Mean F/U: NR
Bierdrager et al. [20]	17	65	Malignant: 17 (100%) CRT: NR, C: NR, R: NR	NR	88.2	88.2	CT thorax Venography	Uncovered: 15 (100%) Symphony	Anticoag: NIL	Imaging: NR Clinical: Patient reported Mean F/U: NR
Sheikh et al. [64]	19	46.4	Benign: 19 (100%)	NR	100	100	NR	Uncovered: 19 (100%) Wallstent, Memotherm, Palmaz, Gianturco-Z	Anticoag: Long term anticoagulation	Imaging: NR Clinical: Patient reported Mean F/U: 28.8 m
Barshes et al. [52]	56	62.6	Malignant: 40 (71%) CRT: NR, C: NR, R: NR Benign: 16 (29%)	NR	100	96.4	Venography	Uncovered: 56 (100%) Palmaz, Wallstent	Anticoag: Heparin 5000 IU Warfarin or clopidogrel	Imaging/Clinical: CXR/USS + assessment at 1, 3, 6, 12 m Mean F/U: NR
Nagata et al. [31]	71	63.4	Malignant: 71 (100%) CRT: NR, C: NR, R: NR	SVC: 71 (100%) + BCV: 17 (24%)	100	87.3	CT thorax Histology Venography	Uncovered: 71 (100%) Spiral-Z, Gianturco-Z, Rosch-Z, Wallstent	Anticoag: Heparin 5000 IU Warfarin 3 m	Imaging: NR Clinical: Patient reported Mean F/U: NR
Lanciego et al. [21]	149	65	Malignant: 149 (100%) CRT: 9, C: 24, R: 4	SVC: 149 (100%) + BCV: 77 (52%)	100	82.6	Venography	Uncovered: 149 (100%) Wallstent	Anticoag: Heparin 5000 IU Oral anticoagulants 6 m	Imaging: NR Clinical: Patient reported Mean F/U: NR
Cho et al. [34]	17	59	Malignant: 17 (100%) CRT: NR, C: NR, R: NR	SVC: 17 (100%) + BCV: 7 (41%) + IJV: 1 (6%)	100	100	CT thorax Venography	Uncovered: 17 (100%) Memotherm, Wallstent, Absolute, Luminexx, Symphony	Anticoag: NIL	Imaging: NR Clinical: Patient reported Mean F/U: NR
Fagedet et al. [39]	164	59.9	Malignant: 164 (100%) CRT: 0, C: 6, R: 3	SVC: 164 (100%) + BCV: 88 (54%)	91.5	90.9	CT angiogram Venography	Uncov/Covered: NR Wallstent, Memotherm, Cordis, Protégé, Strecker	Anticoag: Heparin 3000 IU Aspirin 6 m	Imaging: CT at 6 m, 12 m Clinical: Patient reported Mean F/U: 355.2d
Gwon et al. [25]	73	61.3	Malignant: 73 (100%) CRT: 7, C: 48, R: 1	SVC: 73 (100%) + BCV: 47 (64%)	100	93.2	CT thorax Histology—bronchoscopy, percutaneous needle, excision Venography	Uncovered: 36 (49%) Covered: 37 (51%) ComVi, Zilver	Anticoag: Heparin 5000 IU Aspirin or warfarin 3 m	Imaging: CT at 1 m, 6 m Clinical: Assessment at 1, 3, 6, 9, 12 m Mean F/U: 150d
Maleux et al. [23]	78	64.1	Malignant: 78 (100%) Stenting as first line intervention	SVC: 78 (100%) + BCV: 9 (12%)	100	100	CT thorax Venography	Uncovered: 78 (100%) Zilver	Anticoag: Heparin 5000 IU LMWH 1 m + aspirin	Imaging: NR Clinical: Patient reported Mean F/U: NR
Andersen et al. [44]	25	65	Malignant: 25 (100%) CRT: 25, C: 0, R: 0	NR	96	96	CT thorax Venography	Uncovered: 25 (100%) E-Luminexx, Zilver, Sinus-XL	Anticoag: Heparin 5000 IU Aspirin	Imaging: CT at 3 m Clinical: Patient reported Mean F/U: NR
Cho et al. [24]	40	61.4	Malignant: 40 (100%) CRT: 9, C: 24, R: 1	SVC: 40 (100%) + BCV: 25 (63%)	100	85	CT thorax Histology—bronchoscopy, biopsy Venography	Covered: 40 (100%) ComVi	Anticoag: NR	Imaging: NR Clinical: Patient reported Mean F/U: 175d
Sobrinho and Aguiar [40]	56	59.3	Malignant: 56 (100%) Stenting as first line intervention	NR	87.5	87.5	CT thorax Venography	Uncovered: 49 (100%) Sinus-XL, Smartstent, Wallstent, Express	Anticoag: Heparin 5000 IU LMWH + aspirin	Imaging: NR Clinical: Patient reported Mean F/U: NR
Andersen et al. [44]	12	69	Malignant: 12 (100%) CRT: 12, C: 0, R: 0	NR	91.7	91.7	CT thorax Venography	Uncovered: 12 (100%) Zilver	Anticoag: Heparin 5000 IU	Imaging: CT at 1 m, 3 m Clinical: Patient reported Mean F/U: 2 m
Breault et al. [65]	44	56	Benign: 44 (100%)	NR	88.6	88.6	CT thorax Venography	Uncovered: 40 (100%) Wallstent, Sinus-XL, Luminexx, Smartstent, Express	Anticoag: Heparin 5000 IU	Imaging: NR Clinical: Assessment at 3 m Mean F/U: 1275d
Leung et al. [41]	56	64	Malignant: 56 (100%) CRT: NR, C: NR, R: NR	SVC: 56 (100%) + BCV: 31 (55%)	96.4	91.1	CT thorax Venography	Uncovered: 54 (100%) Wallstent	Anticoag: Heparin	Imaging: NR Clinical: Patient reported Mean F/U: NR
Miazga et al. [66]	112	64	Malignant: 109 (97%) CRT: NR, C: NR, R: NR Benign: 3 (3%)	NR	98.2	98.2	CT thorax Histology Venography	Uncovered: 110 (100%) Epic, Smartstent	Anticoag: NR	Imaging: NR Clinical: Patient reported Mean F/U: NR
Mokry et al. [47]	23	62.5	Malignant: 23 (100%) CRT: 15, C: 3, R: 1	NR	100	95.7	CT thorax Venography	Uncovered: 23 (100%) Sinus-XL	Anticoag: Heparin 2000 IU Heparin 1w	Imaging: NR Clinical: Patient reported Mean F/U: 66d
Büstgens et al. [69]	141	64.6	Malignant: 141 (100%) CRT: 0, C: 57, R: 31	NR	97.9	96.5	CT thorax Histology Venography	Uncov/covered: NR Smartstent, Wallstent, Zilver, Epic	Anticoag: Heparin 5000 IU	Imaging: NR Clinical: Patient reported Mean F/U: NR
Massara et al. [71]	25	65.5	Benign: 25 (100%)	NR	100	100	Venography	Uncovered: 25 (100%) Wallstent, Wallgraft, Express	Anticoag: Dual antiplatelets	Imaging/Clinical: USS + assessment at 1, 3, 6, 12, 18 m Mean F/U: NR
Anton et al. [46]	31	67	Malignant: 31 (100%) CRT: 7, C: 11, R: 0	SVC: 31 (100%) + BCV: 10 (32%)	100	100	CT thorax Venography	Uncovered: 31 (100%) Sinus-XL, Protégé Everflex	Anticoag: Heparin 3000 IU	Imaging: CT Clinical: Patient reported Mean F/U: 184d
Calsina Juscafresa et al. [67]	33	57.6	Malignant: 33 (100%) CRT: NR, C: NR, R: NR	SVC: 33 (100%) + BCV: 20 (61%)	100	84.8	CT angiogram Histology Venography	Uncov/covered: NR Protégé, Wallstent, Express	Anticoag: Heparin 4000 IU	Imaging: NR Clinical: Patient reported Mean F/U: NR
Kuo et al. [68]	12	58.4	Malignant: 12 (100%) CRT: 7, C: 5, R: 0	NR	100	100	CT thorax Histology Venography	Uncovered: 12 (100%) Wallstent	Anticoag: Heparin 3000 IU Clopidogrel	Imaging: CT at 3 m, 6 m, 1y Clinical: Patient reported Median F/U: 11.5 m
Niu et al. [16]	47	NR	Malignant: 47 (100%) CRT: NR, C: NR, R: NR	SVC: 47 (100%) + BCV: 27 (57%)	100	97.9	CT thorax Histology—bronchoscopy, biopsy, oesophageal endoscopy, surgery Venography	Uncovered: 47 (100%) Sinus-XL, Zilver, Luminexx, Smartstent	Anticoag: Heparin 5000 IU Warfarin lifelong	Imaging: CT at 1 m, 3 m, 6 m Clinical: Assessment every 2 m Mean F/U: 6 m
Haddad et al. [26]	59	47	Benign: 59 (100%)	NR	79.7	79.7	CT thorax Venography	Uncov/covered: NR Wallstent, Protégé, Smartstent, Gore Viabahn, iCast	Anticoag: Heparin 5000 IU	Imaging/Clinical: CT + assessment at 3 m, 6 m, 1y Mean F/U: 2.7y (C), 1.8y (U)
Majumdar et al. [22]	10	42.2	Benign: 10 (100%)	NR	80	80	CT thorax Histology Venography	Uncovered: 10 (100%) Wallstent, Palmaz, Cordis, EV3	Anticoag: NR	Imaging: NR Clinical: Patient reported Mean F/U: 3.6y
Karakhanian et al. [72]	28	52.5	Malignant: 18 (64%) CRT: NR, C: NR, R: NR Benign: 10 (36%)	NR	96.4	96.4	CT thorax Venography	Uncov/covered: NR Wallstent, Sinus-XL, Sioxx	Anticoag: Heparin 5000 IU	Imaging: NR Clinical: Assessment for 90d Mean F/U: 90d
Ren et al. [42]	12	64.3	Malignant: 12 (100%) CRT: 1, C: 5, R: 1	NR	100	100	CT thorax Histology Venography	Uncovered: 12 (100%) Sinus-XL, Zilver, Smartstent	Anticoag: Heparin 5000 IU Warfarin	Imaging: CT at 1 m, 3 m, 6 m Clinical: Assessment every 2 m Mean F/U: 4.9 m
Wang et al. [27]	64	61.4	Malignant: 64 (100%) CRT: NR, C: NR, R: NR	SVC: 64 (100%) + BCV: 21 (C), 20 (U) (64%)	100	100	CT thorax Histology—percutaneous biopsy, bronchoscopy, endoscopy Venography	Uncovered: 34 (53%) Covered: 30 (47%) Fluency, Luminexx	Anticoag: Heparin 3d	Imaging/Clinical: Assessment at 1, 3, 6 m Mean F/U: 6.2 m
Wei et al. [17]	16	NR	Malignant: 16 (100%) Stenting as first line intervention	SVC: 16 (100%) + BCV: 4 (25%)	100	100	CT thorax Histology—CT-guided percutaneous biopsy Venography	Uncovered: 16 (100%) Wallstent	Anticoag: Long term anticoagulation	Imaging: NR Clinical: Patient reported Mean F/U: NR

*CRT,* Previous chemoradiotherapy; *C*, previous chemotherapy; *R*, previous radiotherapy; *SVC*, superior vena cava; *BCV,* brachiocephalic veins; *IVC*, inferior vena cava; *NR*, not recorded; *LMWH,* low molecular weight heparin; *M*, malignant; *B,* benign

**Table 2:** Risk-of-bias quality assessment of the 54 included studies according to Newcastle–Ottawa Scale

Study	Comparability				Outcome	Follow-up		Quality
	Representativeness	Selection	Outcome absence pre-intervention	Comparability of cohorts	Assessment of outcome	Appropriate follow-up period	Cohort follow-up achieved	Total (/9)
Dyet et al. [23]	*	*	*	*	*	*	*	9
Gaines et al. [15]	*	*	*	*	*	*	*	9
Crowe et al. [35]	*	*	*	*	*	*		8
Hennequin et al. [57]	*	*	*	*	*	*	*	9
Shah et al. [58]	*	*	*	*	*	*	*	9
Stock et al. [51]	*	*	*	*	*	*	*	9
Oudkerk et al. [18]	*	*	*	*	*	*		8
Gross et al. [32]	*	*	*	*	*	*	*	9
Nicholson et al. [12]	*	*	*	*	*		*	8
Tanigawa et al. [36]	*	*	*	*	*	*	*	9
Qanadli et al. [33]	*	*	*	*	*		*	8
Thony et al. [37]	*		*	*	*	*	*	9
Marcy et al. [59]	*	*	*	*	*	*	*	9
Miller et al. [60]	*		*	*	*	*	*	8
Sasano et al. [29]	*	*	*	*	*	*	*	9
Lanciego et al. [19]	*	*	*	*	*	*	*	9
Smayra et al. [38]	*		*	*	*		*	7
Wilson et al. [70]	*	*	*	*	*	*	*	9
de Gregorio Ariza et al. [53]	*	*	*	*	*	*	*	9
Chatziioannou et al. [61]	*	*	*	*	*	*	*	9
Courtehoux et al. [62]	*	*	*	*	*	*	*	9
Dinkel et al. [49]	*	*	*	*	*	*	*	9
Monaco (2003)	*	*	*	*	*	*	*	9
Kim et al. [63]	*	*	*	*	*	*	*	9
Urreticoechea [30]	*	*	*	*	*	*	*	9
Bierdrager et al. [20]	*	*	*	*	*	*	*	9
Sheikh et al. [64]	*	*	*	*	*	*	*	9
Barshes et al. [52]	*	*	*	*	*		*	8
Nagata et al. [31]	*	*	*	*	*	*	*	9
Lanciego et al. [21]	*	*	*	*	*	*	*	9
Cho et al. [34]	*	*	*	*	*	*	*	9
Fagedet et al. [39]	*	*	*	*	*	*	*	9
Gwon et al. [25]	*	*	*	*	*	*	*	9
Maleux et al. [23]	*	*	*	*	*	*	*	9
Andersen et al. [44]	*	*	*	*	*	*	*	9
Cho et al. [24]	*	*	*	*	*	*	*	9
Sobrinho and Aguiar [40]	*	*	*	*	*	*	*	9
Andersen et al. [44]	*	*	*	*	*	*	*	9
Breault et al. [65]	*	*	*	*	*	*	*	9
Leung et al. [41]	*	*	*	*	*	*	*	9
Miazga et al. [66]	*	*	*	*	*	*	*	9
Mokry et al. [47]	*	*	*	*	*	*	*	9
Büstgens et al. [69]	*	*	*	*	*	*	*	9
Massara et al. [71]	*	*	*	*	*	*	*	9
Anton et al. [46]	*	*	*	*	*	*	*	9
Calsina Juscafresa et al. [67]	*		*	*	*	*	*	8
Kuo et al. [68]	*	*	*	*	*	*	*	9
Niu et al. [16]	*	*	*	*	*	*	*	9
Haddad et al. [26]	*		*	*	*	*	*	8
Majumdar et al. [22]	*		*	*	*	*	*	8
Karakhanian et al. [72]	*	*	*	*	*	*	*	9
Ren et al. [42]	*	*	*	*	*	*	*	9
Wang et al. [27]	*	*	*	*	*	*	*	9
Wei et al. [17]	*	*	*	*	*			7

All studies scored between 7 and 9, indicating low risk of bias and high quality

All studies relied on clinical criteria to determine need for intervention, as well as pre-operative imaging to denote the nature of the obstruction. Five papers selected only patients presenting with significant stenosis, ranging between 75 and 90% [18–22]. One study categorised patient population by level of stenosis into high (> 80%), moderate (50–80%), and low grade (30–50%) [23].

Follow-up protocol was heterogenous among the included studies. Where prospectively specified, it involved regular imaging and clinical assessment in 9/54 studies or imaging only in 10/54 studies. The remaining studies relied on patients self-reporting symptoms. Length of follow-up was variable, with mean follow-up lengths ranging from 2 months to 3 years. All studies followed patients up where possible until death or study endpoint.

### SVCS Pathology

Malignant or benign pathology was the exclusive cause of SVCS in 39/54 studies and 6/54 studies respectively. The remaining 9 studies did not discriminate by pathology. Of the 2237 patients in which SVCS pathology was reported, 222 had benign and 2015 had malignant pathology. The most frequent malignant pathologies included non-small cell and small cell bronchial carcinoma, as well as lymphadenopathies or invasion from extra-mediastinal primary tumours. Of the 6 studies that reported outcomes in benign SVCS, the primary pathology in 5 of these studies was indwelling medical devices, of which 1 included patients on dialysis. The primary pathology in the remaining study was fibrosing mediastinitis.

### Stent Type

The type of stent used was reported in 50/54 studies, comprising 1795 patients. Uncovered stents were exclusively used in 47/50 studies. One study exclusively used covered stents [24], and three studies used both covered and uncovered stents in direct subgroup comparison [25–27]. The rationale for using covered stents in these papers was their anecdotal use in several published case reports of recurrent SVCS after uncovered stent placement or iatrogenic injury of the SVC [24–27]. The total number of patients receiving uncovered or covered stents was 1688/1795 and 107/1795.

### Intra-procedural Anticoagulation

The use of intra-procedural anticoagulation was documented in 42/54 studies, comprising 1861 patients. Fixed doses of heparin injected before stenting were documented in 34/42 studies, with doses of 2000–5000 IU used. The most common dose used was 5000 IU in 28/34 studies. Use of heparin in the post-operative period at unspecified doses or frequency was documented in 8/42 studies.

### Long-Term Anticoagulation

The use of long-term anticoagulation was documented in 27/54 studies, comprising 1197 patients. Regimens used were heterogenous in medication and duration of treatment. Where specified, regimens used more than once included 3 months of warfarin in 4 studies [28–31] or 1 month of dual antiplatelet therapy in 2 studies [32, 33]. Anticoagulation medications used included warfarin, aspirin, heparin, antiplatelets and their combinations. Three studies comprising 142 patients stated that neither intra-procedural nor long-term anticoagulation was attempted [3, 20, 34].

### Intra-procedural Thrombolysis

The use of intra-procedural thrombolysis before stenting was documented in 19/54 studies, comprising 727 patients. In 16/19 studies, thrombolysis was only used for cases in which thrombosis above the stent was too severe to navigate across. In 3/19 studies, thrombolysis was used for all patients to prevent intra-stent thrombosis in follow-up [18, 35, 36]. The pharmacological agent used was specified in 16/19 studies, with urokinase, recombinant tissue plasminogen activator and streptokinase used in 8, 5 and 3 studies respectively. Mechanical thrombolysis, relying on thromboaspiration, fragmentation or crushing the thrombus against the vessel wall, was used in 3/19 studies [37–39].

### Previous Treatments Attempted

Stenting was attempted as the first-line procedure for treatment of malignant SVCS in 4/54 studies, comprising 202 patients [17, 20, 22, 40]. One further study retrospectively investigated a cohort of 56 malignant SVCS patients, with 33 patients undergoing stenting at initial presentation before chemoradiotherapy and 23 only after the failure of chemoradiotherapy [41].

### Technical Success

**Fig. 2:**
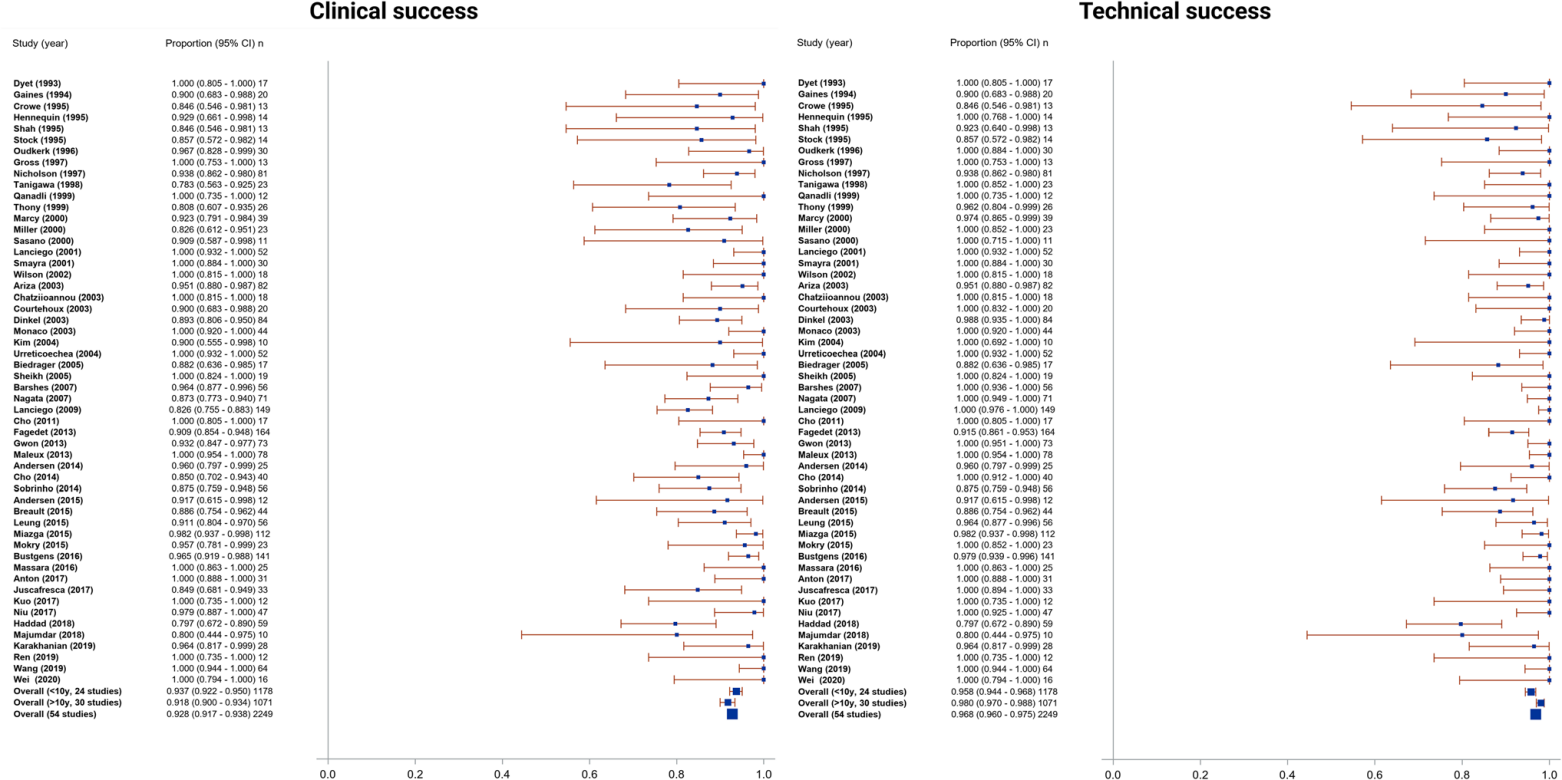
Forest plots showing **a** technical and **b** clinical success plots. Pooled technical success rate was 96.8% (95% CI 96.0–97.5%, range 79.7–100%, *I*^2^ = 0). Pooled clinical success rate was 92.8% (95% CI 91.7–93.8%, range 78.3–100%, *I*^2^ = 0)

**Fig. 3:**
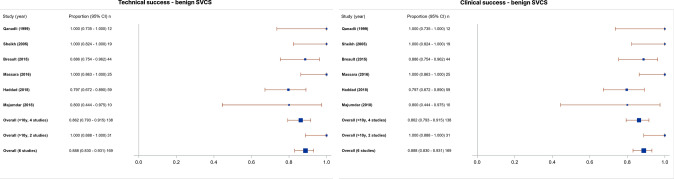
Forest plots showing **a** technical and **b** clinical success plots for 6 studies investigating benign SVCS alone. Pooled technical success rate and clinical success rate were 88.8% (95% CI 83.0–93.1%)

The technical success rate was 96.8% (95% CI 96.0–97.5%), with a range of 79.7–100% and *I*^2^ = 0 indicating no heterogeneity (Fig. 2). The technical success rate for studies investigating benign SVCS alone was 88.8% (95% CI 83.0–93.1%) (Fig. 3). Most studies described technical success as navigation and successful deployment and expansion of the stent across the obstruction or stenosis, with evidence of flow restoration on post-intervention venography. Further requisites for technical success where specified included a final pressure gradient < 10 mmHg in 4 studies [16, 24, 25, 42] and < 50% residual stenosis in 3 studies [22, 43, 44]. Two studies used the Society of Interventional Radiologists (SIR) definition of technical success; complete coverage of the obstruction, with overlapping margins of 1 cm on either side and residual stenosis < 30% [45–47].

### Clinical Success

The clinical success rate was 92.8% (95% CI 91.7–93.8%), with a range of 78.3–100% and *I*^2^ = 0 indicating no heterogeneity (Fig. 2). The clinical success rate for studies investigating benign SVCS alone was 88.8% (95% CI 83.0–93.1%) (Fig. 3). All studies except two described clinical success as acute improvement in symptoms, whether partial or complete, measured through patient description of symptoms or the Kishi scoring system [48]. One study further defined clinical failure as persistence of at least 2 of the cardinal symptoms of SVCS; prominent veins, facial oedema, plethora, dizziness, headaches and dyspnoea [49]. Dyspnoea was exempted from consideration as a symptom of clinical improvement in most studies, as it is a common symptom of underlying pulmonary disease and is frequently found in patients presenting with tumour invasion into the bronchus or pulmonary vessels. The other study defined clinical success as < 10 mmHg pressure gradient between ends of the stent after insertion [27].

### Complications

Following the CIRSE complication [50], complications are presented in Table 3. The average complication rate was 5.78% (SD = 9.3182), with a range of 0–53.8%. No complications were reported in 25/54 studies. The overall 24-h mortality rate was 0.006%. The most frequent cause of mortality was rupture of the SVC leading to cardiac tamponade. The most frequent complications above Grade 3 reported were bleeding events while on long-term anticoagulation or antiplatelet therapy, pulmonary oedema and thromboembolic events. The most frequent complications below Grade 3 reported were stent migration, localized pain and puncture site haematoma.

**Table 3:** Minor and major complications by Cardiovascular and Interventional Radiological Society of Europe (CIRSE) classification

*Grade 1*		*Grade 3*	
Localised pain	12	Haemopericardium	4
Puncture site haematoma	11	Sepsis	3
Fever	7	Arterial injury	2
Tachypnoea	2	Lower limb cellulitis/phlebitis	2
*Grade 2*		*Grade 4*	2
Stent migration	17	Bleeding event on anticoagulation	19
Arrhythmia—SVT (4), VT (1), bradycardia (1)	6	Pulmonary embolism/DVT	8
Haemoptysis/haematemesis	6	Hoarseness due to laryngeal nerve damage	3
Transiently impaired venous drainage	1	*Grade 6*	3
*Grade 3*		Mortality in 24 h—tamponade (5), unknown (4), MI (1), PE (1), HF (1), haemopericardium (1)	13
Pulmonary oedema	10		
Cardiac tamponade due to iatrogenic SVC perforation	7		

*SVT,* supraventricular tachycardia; *VT,* ventricular tachycardia; *DVT,* deep venous thrombosis*; MI,* myocardial infarction; *PE,* pulmonary embolism*; HF,* heart failure

### Recurrence and Re-interventions

Primary patency was reported in 19/54 studies, comprising 906 patients, while secondary patency was reported in 20/54 studies, comprising 1117 patients. Primary and secondary patency ranged from 65 to 92% and 75–100% respectively. Primary patency was defined as continued stent patency without re-intervention at study endpoint, while secondary patency included those requiring re-interventions. Four studies separately defined primary patency as the time interval from procedure to re-intervention, ranging from 83 days to 31.3 months [16, 22, 27, 51].

**Fig. 4:**
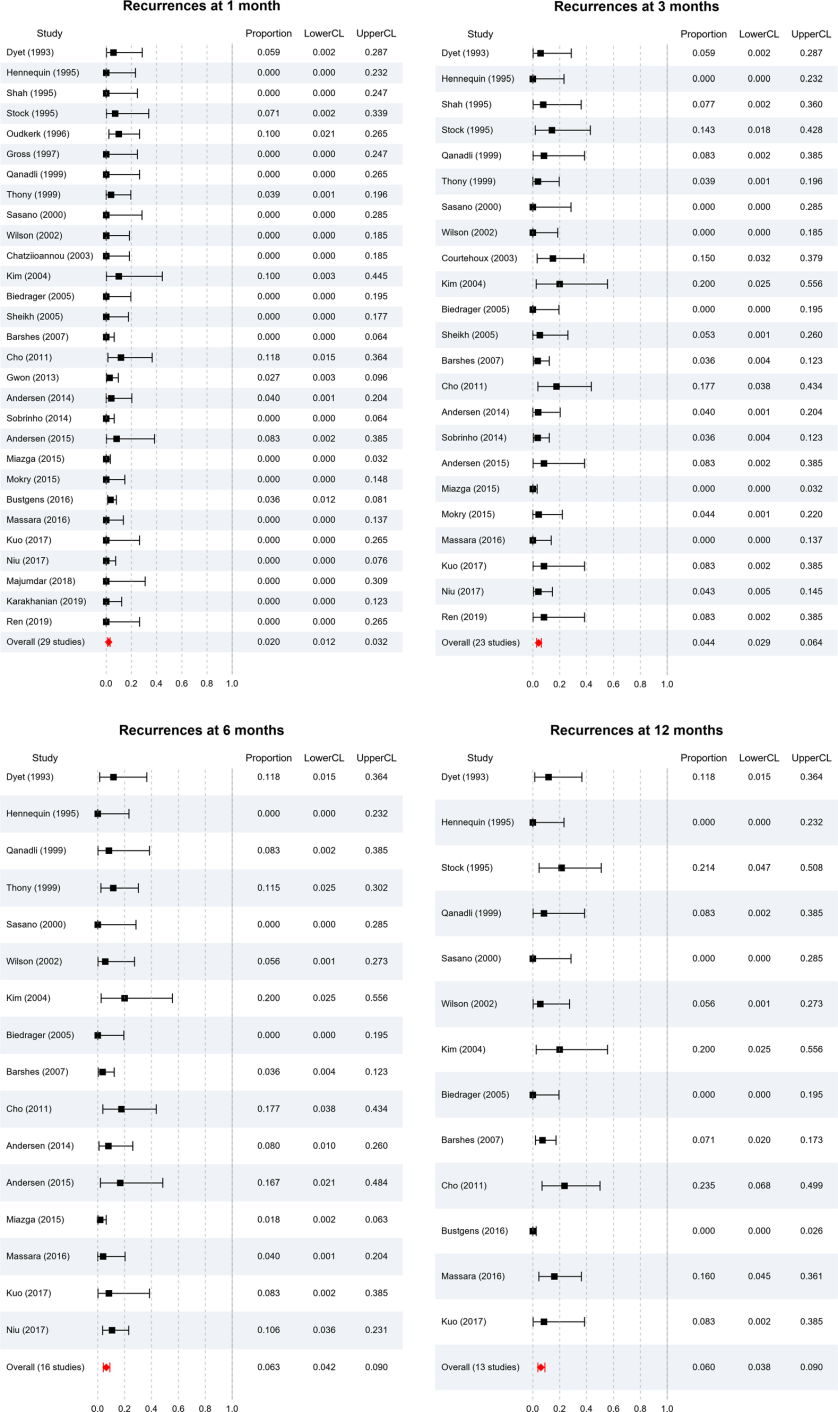
Forest plots showing recurrence rates at **a** 1 month, **b** 3 months, **c** 6 months and **d** 12 months. Pooled patency remained above 90% for the first year (98.0%, 95.6%, 93.7% and 94.0% at 1, 3, 6 and 12 months respectively. At all timepoints, *I*^2^ = 0

Recurrence rate of symptoms in follow-up was reported in 29/54, 23/54, 16/54 and 13/54 studies at 1, 3, 6 and 12 months respectively (Fig. 4). From these studies, pooled patency for endovascular stenting in both benign and malignant SVCS remained above 90% for the first year (98.0%, 95.6%, 93.7% and 94.0% at 1, 3, 6 and 12 months respectively). At all timepoints, *I*^2^ = 0 indicating no heterogeneity. In order of frequency, the cause of recurrence was intra-stent thrombosis, tumour overgrowth above or below the stent, or tumour ingrowth through the stent. The average re-intervention rate was 9.11% (SD = 11.190), with a range from 0 to 60%. No re-interventions were required in 17/54 studies. Re-interventions performed included balloon dilatation, thrombolysis and further stenting.

One study found primary patency to be significantly longer in patients with malignant SVCS compared to SVCS secondary to haemodialysis [38]. Three studies found covered stent use to be significantly associated with lower rates of stent occlusion in follow-up, lower rates of symptom recurrence and longer primary and secondary patencies [25–27].

### Survival

Median and mean survival were reported in 12/54 studies and 10/54 studies respectively, of which all but one study comprised patients with malignant pathology. The average median survival was 4.74 months (SD = 3.3602), with a range of 1–13 months. The average mean survival was 4.71 months (SD = 1.4235), with a range of 2.49–6.70 months. One study found survival was significantly longer in patients in whom stenting was attempted as a first-line procedure [41]. Another study found survival was significantly longer in patients who underwent subsequent chemotherapy, or chemoradiotherapy, as compared to patients who did not receive further treatment [21].

## Discussion

This systematic review and meta-analysis confirm the efficacy and safety of endovascular intervention in SVCS, with high technical and clinical success rates of 96.8% and 92.8% respectively, patency remaining above 90% for the first year, and low complication and re-intervention rates. These results parallel current perceptions of SVCS among clinicians and correspond with the existing literature, which posits a technical success rate above 80% and clinical success rate above 90% [4, 7].

There is a relative paucity in research into benign SVCS, and this is reflected in the balance of studies investigating benign SVCS in this review. Of the studies investigating benign SVCS in this review, pooled technical and clinical success rates were identical at 88.8%. Several studies report differences in patency between patients with benign and malignant SVCS [39, 52, 53]. These results are however mixed and did not reach statistical significance, aside from a study by Smayra et al. which found primary patency to be significantly longer in malignant SVCS compared to haemodialysis-associated SVCS. Given the shift in aetiology of benign SVCS towards indwelling medical devices, and the resultant predicted increase in benign SVCS incidence [5], it is critical more research is directed into these pathologies.

Patients with benign SVCS also have higher life expectancies and survival [5, 10, 54]. The impact of declining stent patency with time, subsequent risk for re-intervention and need for long-term anticoagulation may hence be greater. Sfyroeras and colleagues performed a systematic review of 9 studies investigating benign SVCS and found pooled patencies of 90.7%, 71.2% and 48% at 1 month, 12 months and 36 months respectively, with 26.9% of patients requiring re-intervention [7]. The risks and benefits of stenting as a palliative procedure in malignant SVCS or therapeutic procedure in benign SVCS should be considered separately.

The use of covered stents was found in three studies to significantly improve outcomes [25–27]. Gwon et al. investigated 73 patients with malignant SVCS and reported significantly higher cumulative patencies across the first year between covered and uncovered stents [25]. This was corroborated in further studies of both benign and malignant SVCS [26, 27]. These three studies were however three of only four studies in this review to use covered stents, emphasizing the need for further research into the role of covered stents in the future.

Despite the growing body of evidence for stenting in SVCS, there is little evidence for a standardised anticoagulation regimen, both intra-procedurally and in follow-up. It has further not been proven that anticoagulation leads to improved outcomes. Ratzon et al. retrospectively investigated 183 malignant SVCS patients and found no statistically significant difference in intra-stent thrombosis in follow-up or survival associated with anticoagulation [55]. Similarly, Haddad et al. did not find a statistically significant difference in symptom recurrence, mean percent stenosis in follow-up, time to return of symptoms or primary patency in a population of 58 benign SVCS patients [56]. Given that adverse events on anticoagulation represent a significant proportion of complications post-procedure, further research is needed to identify if anticoagulation is necessary, and the ideal regimen.

The role of intra-procedural thrombolysis also requires further exploration. Fagedet et al. identified thrombosis as a risk factor significantly doubling the risk of symptom recurrence (HR 2.60), with this risk removed by using intra-procedural thrombolysis [39]. There is however little research published examining the impact of thrombolysis on long-term outcomes, or whether it should be used as a prophylactic measure against stent thrombosis in follow-up.

Stenting has largely replaced radiation therapy as the first line procedure for managing malignant SVCS, due to its immediate nature, high success rate, and the retained possibility to attempt alternative therapies [4, 10, 12]. The only study to specifically examine the impact of stenting as a first-line procedure was a retrospective study of 56 malignant SVCS patients by Leung et al. [41]. They found that patients who received stenting at initial presentation had significantly increased survival over patients who received stenting after the failure of traditional treatments, but found no significant difference in success rate, procedure time, symptom relief, complication rate or re-intervention rate. There are furthermore no published studies comparing endovascular intervention directly with chemotherapy, radiotherapy or surgical intervention alone in a randomized controlled trial.

A key limitation of this review is the lack of randomised controlled trials or prospectively designed studies with clearly specified follow-up strategies. Most of the studies included were retrospective, single-centre studies, raising the risk of selection or publication bias and possible overestimation of results. The inconsistency in following up patients and proportion of patients lost to follow-up limit review of long-term outcomes. This is further compounded by the short life expectancies or survival of malignant SVCS patients. Definitions of technical success, clinical success and primary and secondary patency varied across studies and guidelines of learned societies were not strictly followed, limiting the utility of meta-analysis. Further subgroup meta-analysis by SVCS pathology or additional therapies given was not performed due to the small proportion of data on such patients, limiting the applicability of these findings.

In conclusion, this systematic review and meta-analysis confirm endovascular stenting as a safe and effective therapeutic option for SVCS of all pathologies, with high technical and clinical success rates, as well as low complication and recurrence rates. Our study also consolidates current evidence for the impact of procedural considerations, such as stent type, use of anticoagulation and intra-stent thrombolysis. More research of higher methodological quality, such as randomised controlled trials or larger multi-centre studies, is needed to better elucidate the scope of efficacy of stenting, as well as the patients to which stenting could most benefit.
